# Rare Ocular Phenomenon: A Case of Ping-Pong Gaze

**DOI:** 10.7759/cureus.47767

**Published:** 2023-10-26

**Authors:** Gajanan Chavan

**Affiliations:** 1 Emergency Medicine, Jawaharlal Nehru Medical College, Wardha, IND

**Keywords:** pres, gaze abnormalities, cerebral infarction, bilateral cerebral involvement, ping-pong gaze

## Abstract

Ping-pong gaze, a rare neurological phenomenon characterized by pendular, rhythmic oscillations of the eyes, is explored in a unique clinical context. A 40-year-old male with a history of chronic pancreatitis, type two diabetes mellitus, and chronic alcoholism presented with hypoglycemic coma and synchronous horizontal eye movements indicative of ping-pong gaze. Radiological assessments hinted at cerebral infarction or posterior reversible encephalopathy syndrome (PRES). Despite aggressive intensive care management, the patient’s condition deteriorated, highlighting the intricate challenge of managing hypoglycemic coma amidst brain pathology, metabolic disorders, and chronic alcoholism. This exceptional case underscores the need for prompt recognition of ping-pong gaze, comprehensive care, and further investigation into its underlying medical conditions.

## Introduction

Ping-pong gaze is an intriguing and rare neurological phenomenon that captures the attention of clinicians and researchers alike. This distinctive ocular sign is characterized by involuntary, pendular, rhythmic oscillations of the eyes, often resembling the back-and-forth movement of a ping-pong ball. Ping-pong gaze is not a single, isolated entity but a manifestation of various neurological conditions affecting the neural pathways responsible for controlling eye movements. Its rarity has limited its documentation in the medical literature, making it a captivating subject of study [1].

While the exact pathophysiology behind the ping-pong gaze remains a subject of ongoing research, its appearance can be a critical diagnostic clue, prompting clinicians to investigate and explore the underlying causes. These causes may range from brainstem lesions to hereditary syndromes. The case of ping-pong gaze described in this report holds particular significance due to its rarity and association with a unique set of underlying conditions, including chronic pancreatitis, type two diabetes mellitus, and chronic alcoholism [2].

## Case presentation

A 40-year-old male was brought to the emergency department by his relatives with complaints of loss of consciousness for one day. The patient was conscious the previous night but was found unconscious the next morning. He had a five-year history of chronic pancreatitis and is not currently on medication. Additionally, he was a chronic alcoholic and diabetic, for which he was on daily insulin and metformin. He also had a history of hospitalization on multiple occasions due to hypoglycemic episodes.

Upon arrival, his bedside glucose testing showed a critically low value of 36 mg/dl, prompting immediate intervention with 200 ml of 25% dextrose and thiamine 500 mg. The patient presented with a heart rate of 150 per minute, blood pressure at 120/90 mmHg, oxygen saturation of 88% on room air, and was afebrile. Bilateral basal crepitations with equal air entry were noted on chest examination, and neurological examination revealed a low Glasgow Coma Scale (GCS) score of five. His GCS components included an eye response score of two (he opened his eyes to pain), a verbal response score of one (absent verbal response), and a motor response score of two (abnormal extensor response to pain stimulus). Notably, the patient exhibited ping-pong gaze, characterized by synchronous, horizontal, oscillatory eye movements (Video 1). No movements in the vertical plane or any rotatory movements were observed. Bilateral pupils were equal in size but non-reactive to light, and plantar reflexes were absent. Given the low GCS score, the patient was promptly intubated and placed on a mechanical ventilator.

**Video 1:** Ping-pong gaze

**Figure 1:**
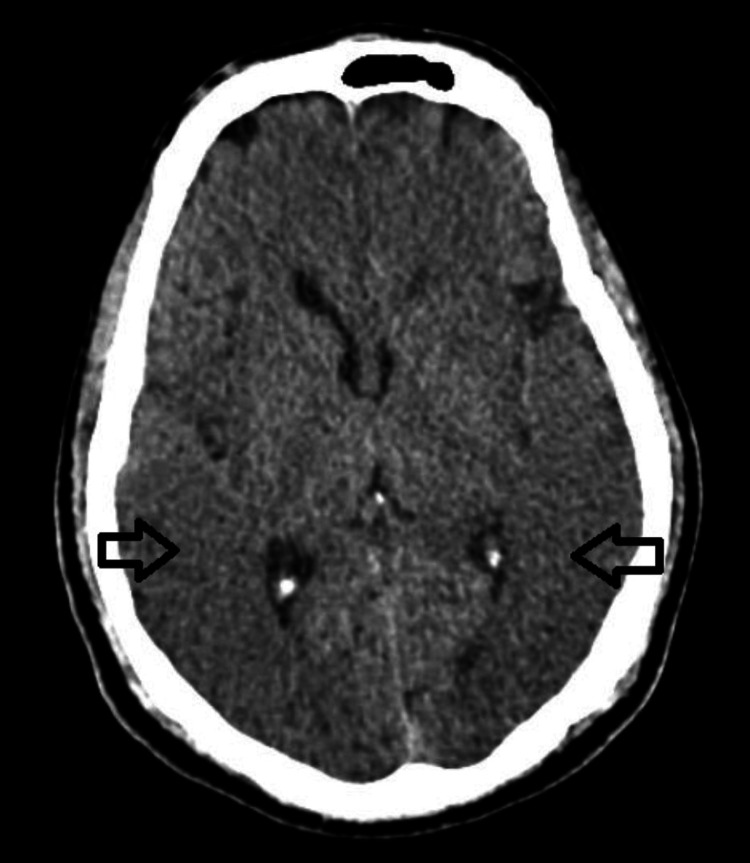
Non-contrast computerized tomography (CT) brain scan; arrows indicate the loss of gray-white matter differentiation with effacement of sulcal gyral spaces in the bilateral temporal and parietal occipital lobe

A computerized tomography (CT) scan of the brain revealed a loss of gray-white matter differentiation with effacement of sulcal gyral spaces in the bilateral temporal and parietal occipital lobe (Figure 1). The CT report suggested possible differentials, including acute infarct or posterior reversible encephalopathy syndrome (PRES), recommending further evaluation with magnetic resonance imaging (MRI). However, due to the patient’s unstable condition and financial constraints, an MRI was not pursued, and he was admitted to the intensive care unit (ICU).

Apart from a raised serum urea level of 71 mg/dl and an elevated hemoglobin A1C (HbA1C) value of 10.03, the patient’s initial blood labs, including complete blood counts, liver function tests, creatinine, electrolytes, ammonia, and urine analysis, exhibited normal values. Abdominal sonography revealed no abnormalities. A diagnosis of severe hypoglycemia-induced coma was established. The patient received conservative treatment, including antibiotics, low molecular weight heparin, antacids, mannitol, insulin, and routine ICU care. Despite aggressive medical management, the patient’s prognosis remained grim, ultimately leading to his unfortunate demise after several days.

## Discussion

Ping-pong gaze, an intriguing and rare neurological phenomenon characterized by rhythmic oscillations of the eyes, has been predominantly documented in isolated case reports within the medical literature [1,2]. While this distinctive ocular sign is often associated with extensive bilateral hemispheric involvement, a study by Larmande et al. challenged this conventional understanding by reporting a case of ping-pong gaze in a patient with a localized lesion in the crus cerebri, devoid of significant hemispheric involvement [3]. This intriguing finding suggests that the etiology of the ping-pong gaze may extend beyond hemispheric lesions, warranting further investigation into its underlying mechanisms.

The precise cause and pathophysiology of ping-pong gaze remain elusive. Nevertheless, it has been observed in a variety of clinical contexts, including acute ischemic stroke, hypoxic-ischemic encephalopathy, severe monoamine oxidase inhibitor toxicity, combined intoxication with multiple medications, bacterial meningitis, hypoglycemic encephalopathy, cerebellar hemorrhage, severe bihemispheric brain injury secondary to intravascular large B-cell lymphoma, and refractory focal status epilepticus, often emerging in the post-seizure state [1-10]. Notably, ping-pong gaze has been consistently linked to high mortality rates, although neurological remission is not uncommon [2].

In our presented case, a 40-year-old male with a complex medical history, including chronic pancreatitis, type II diabetes mellitus, and chronic alcoholism, exhibited a ping-pong gaze in the context of a severe hypoglycemic episode. The most likely causes of the observed ping-pong gaze, in this case, are cerebral infarct or posterior reversible encephalopathy syndrome (PRES), as suggested by the CT report, and hypoglycemic encephalopathy, as indicated by the patient’s history and clinical findings. Importantly, our patient presented with extensive bilateral cerebral involvement, aligning with the typical presentation in most reported cases [1,2]. Cerebral infarct associated with ping-pong gaze has been reported in multiple studies while hypoglycemic encephalopathy has also been linked to ping-pong gaze, as reported by Ali and Haines [2,8]. Notably, no existing literature appears to associate PRES with ping-pong gaze. However, it should be acknowledged that the CT report suggested these possibilities without confirmation, highlighting the potential value of an MRI for a definitive diagnosis.

The patient’s prognosis remained bleak despite aggressive medical interventions, leading to an unfortunate outcome. This case serves as a poignant reminder of the formidable challenges posed by prolonged hypoglycemia-induced coma in individuals with underlying metabolic disorders exacerbated by the burden of chronic alcohol abuse. While ping-pong gaze itself is an unusual clinical finding, its occurrence in the context of this unique set of conditions hypoglycemia, type two diabetes mellitus, chronic alcoholism, and chronic pancreatitis illuminates this case’s exceptional rarity and complexity.

## Conclusions

Given the high mortality associated with this phenomenon, this case underscores the critical importance of recognizing the clinical sign - ping-pong gaze, and the necessity for aggressive treatment to address the underlying pathology. Furthermore, this case contributes to the limited medical literature on conditions associated with ping-pong gaze and highlights the significance of imaging studies in its diagnosis. It also sheds light on the potential association between brain pathology and metabolic disorders such as diabetes, chronic alcohol use, and chronic pancreatitis with neuro-ocular manifestations like ping-pong gaze. Further research is warranted to unveil the intricate mechanisms underpinning this enigmatic phenomenon and to enhance diagnostic and management strategies for patients presenting with ping-pong gaze.
